# Social Support in Adulthood Buffers the Effects of Adverse Childhood Experiences on Mortality Risk

**DOI:** 10.1093/geroni/igaf122.2998

**Published:** 2025-12-31

**Authors:** Meredith Willard, Ryan Best, Nicholas Turiano

**Affiliations:** West Virginia University, Morgantown, West Virginia, United States; West Virginia University, Morgantown, West Virginia, United States; West Virginia University, Morgantown, West Virginia, United States

## Abstract

Exposure to Adverse Childhood Experiences (ACEs) is associated with life-long consequences that can negatively affect aging, such as increased risk for health conditions and premature mortality (Anda et al., 2006; Brown et al., 2009). However, supportive social networks can provide buffering effects for certain individuals, which may be critical for those who experience ACEs. However, more evidence suggests that strainful social networks can amplify the negative consequences of ACEs (Rook, 1990). Thus, we utilized data from the Midlife in the United States (MIDUS) study (N = 6,150), to examine if latent profiles of social support and strain buffered/amplified the effect of ACEs on 26 year mortality risk in adulthood. In 1995, respondents (aged 25-74) answered retrospective reports of childhood adversity and social support and strain questions. A latent profile analysis revealed a 5-class solution was most appropriate (AIC: 52868.923; Entropy: 0.828). Next, interactions were created between ACEs and each of the 5 classes to predict mortality risk via a Cox Proportional Hazards model. Although higher ACEs predicted a significantly increased mortality risk (p < .05), being in a class characterized by high support and low strain, ‘Optimal network’ class, or average levels of support and strain, the ‘Average network’ class, mitigated this risk. This study provides evidence that ACEs continue impacting health well into older adulthood, and that using a person-centered approach to capture broader aspects of social support/strain is useful in understanding the protective effects these social relationships exert throughout adulthood.

